# Anesthesia/surgery-induced learning and memory dysfunction by inhibiting mitophagy-mediated NLRP3 inflammasome inactivation in aged mice

**DOI:** 10.1007/s00221-023-06724-4

**Published:** 2023-12-26

**Authors:** Jian Lu, Youming Zong, Xiaoyan Tao, Hongyu Dai, Jiale Song, Hongmei Zhou

**Affiliations:** 1 Department of Anesthesiology, The Second Hospital of Jiaxing, The Second Affiliated Hospital of Jiaxing University, Jiaxing City, Zhejiang Province China; 2 Department of Nursing, The Second Hospital of Jiaxing, The Second Affiliated Hospital of Jiaxing University, Jiaxing City, Zhejiang Province China

**Keywords:** Postoperative cognitive deficit, Mitophagy, Aged mice, NLRP3, Neuroinflammation

## Abstract

Postoperative cognitive dysfunction (POCD) is a common postoperative complication, not only affects the quality of life of the elderly and increases the mortality rate, but also brings a greater burden to the family and society. Previous studies demonstrated that Nod-like receptor protein 3 (NLRP3) inflammasome participates in various inflammatory and neurodegenerative diseases. However, possible mitophagy mechanism in anesthesia/surgery-elicited NLRP3 inflammasome activation remains to be elucidated. Hence, this study clarified whether mitophagy dysfunction is related to anesthesia/surgery-elicited NLRP3 inflammasome activation. POCD model was established in aged C57BL/6 J mice by tibial fracture fixation under isoflurane anesthesia. Morris Water Maze (MWM) was used to evaluate learning and memory abilities. We found that in vitro experiments, lipopolysaccharide (LPS) significantly facilitated NLRP3 inflammasome activation and mitophagy inhibition in BV2 cells. Rapamycin restored mitophagy and improved mitochondrial function, and inhibited NLRP3 inflammasome activation induced by LPS. In vivo experiments, anesthesia and surgery caused upregulation of hippocampal NLRP3, caspase recruitment domain (ASC) and interleukin-1β (IL-1 β), and downregulation of microtubule-associated protein light chain 3II (LC3II) and Beclin1 in aged mice. Olaparib inhibited anesthesia/surgery-induced NLRP3, ASC, and IL-1β over-expression in the hippocampus, while upregulated the expression of LC3II and Beclin1. Furthermore, Olaparib improved cognitive impairment in older mice. These results revealed that mitophagy was involved in NLRP3 inflammasome-mediated anesthesia/surgery-induced cognitive deficits in aged mice. Overall, our results suggested that mitophagy was related in NLRP3 inflammasome-induced cognitive deficits after anesthesia and surgery in aged mice. Activating mitophagy may have clinical benefits in the prevention of cognitive impairment induced by anesthesia and surgery in elderly patients.

## Introduction

POCD is a neurological complication following anesthesia and surgery in elderly patients, mainly affects cognition, learning, and memory (Berger et al. 2015; Steinmetz and Rasmussen 2016; Holmgaard et al. 2019). The incidence of POCD is 41.4% at discharge and 12.7% at 3 months following anesthesia and non-cardiac surgery (Evered et al. 2018). It can last for days, months, or even years, significantly affecting recovery and increasing morbidity and mortality after surgery (Steinmetz et al. 2009; Bilotta et al. 2010; Quan et al. 2019).

Although POCD is an important clinical problem, the pathogenesis of POCD is not well understood. Furthermore, elucidating its pathogenesis is helpful to prevent the occurrence of disease. Several studies have revealed that tissue damage caused by surgery activates the peripheral immune system and promotes an inflammatory response, leading to neuroinflammation (Liu et al. 2018; Wang et al. 2022; Subramaniyan and Terrando 2019). Neuroinflammation has been suggested to play a critical role in the development of POCD (Wei et al. 2019; Li et al. 2022). Wang et al. showed that isoflurane induced age-related hippocampal neuroinflammation via NLRP3 inflammasome activation (Wang et al. 2018). NLRP3 inflammasome causes cognition deficits in age-related neuroinflammation (Youm et al. 2013) and neurodegeneration such as in Alzheimer’s disease (Heneka et al. 2013; Goldmann et al. 2013).

Based on those facts, the NLRP3 inflammasome plays an important role in the development of inflammatory response. Mitophagy can inhibit the activation signal of NLRP3 inflammasome by removing damaged mitochondria (Xu et al. 2019), and regulate inflammatory response to avoid excessive inflammatory response to the body’s damage (He et al. 2019; Chang et al. 2022). Therefore, in this study, we explored whether mitophagy activation could inhibit the neuroinflammation mediated by NLRP3 inflammasome and ameliorate anesthesia/surgery-elicited cognitive decline.

## Materials and methods

### Ethics statement

This study was approved by the Animal Care and Use Committee of the Second Affiliated Hospital of Jiaxing University (Permit Number: JXEY-2020SZ034). All animal procedures complied with the NIH Laboratory Animal Care and Use Guidelines Statement. Efforts were made to reduce the pain caused by surgery and to reduce the total number of animals used.

### Animals

16-month-old male C57BL/6J mice were provided by the Shanghai Institute of Family Planning Science. They were kept under 12-h light–dark cycle and controlled room conditions (24 ± 2 °C; 50 ± 10% humidity). The mice were free to eat food and water. All the mice were acclimated for 7 days before starting the experiment.

### Tibial fracture fixation

Intramedullary fixation of tibial fractures was performed under isoflurane anesthesia (2.0% isoflurane in 0.30 fraction of inspiration O_2_ (FiO_2_) (Feng et al. 2017). After making a skin incision just below the knee, exposed the tibia and inserted a 0.3 mm needle into the medullary cavity. Then, breaked the tibia at its midpoint. Third, 0.1% lidocaine was used around the incision for analgesia and 5–0 Vicryl suture was used to close the wound. During the whole experiment, the temperature of mice was controlled between 36 and 37 °C with a warming pad (ATC-200; World Precision Instruments, Sarasota, Florida USA). After surgery, the mice were spontaneously resuscitated. MWM was used to test learning and memory abilities on the third day after surgery.

### Behavioral testing

MWM was used to assess learning and memory abilities (Vorhees and Williams 2006). In the previous study, mice rested for 2 days after surgery (Su et al. 2011). Twelve mice in each group underwent behavioral tests. The MWM with a white circular pool, 110 cm in diameter and 60 cm deep, a circular platform was hidden at 1.0 cm beneath the surface of water, a platform, 10 cm in diameter. The pool was filled with opaque milky water (23–25 C°) to a depth of 35 cm. The pool was surrounded by invariable visual cues which were not changed till the end of the experiment. The MWM test results of all subjects were monitored and tracked by television camera (HIK VISION Co., Ltd., Hangzhou, China) mounted overhead.

The MWM test included training trials and probe trials. The training trials were performed for 4 days. Each day, mice were put into the maze at the different points. Once the mouse found the platform, mice were allowed to rest on the platform for 30 s. When the mouse did not find the platform within 60 s, the mice were guided to the platform and rest for 30 s. MWM software was used to calculate latency to reach the platform, time spent in each quadrant and swimming speed (RWD Co., Ltd., Shenzhen, China). The probe trials were completed on the 7th day after operations. In probe test, the platform was removed and mice swam for 60 s, and recorded time spent in each quadrant (Gao et al. 2021).

### Cell cultured and treatment

BV2 cell lines were purchased from Procell Life Science & Technology Co., Ltd (China) and cultured in DMEM/HIGH medium containing 10% fetal bovine serum (FBS), in a humidified atmosphere of 5% CO_2_ at 37 °C. The BV2 cells were plated into 6-well plates and treated on the first day after cell attachment, the corresponding treatments were carried out, respectively: control, LPS (1 μmol/L), and LPS + Rapa (0.1 μmol/L). After cultivation for 24 h, cells were collected for immunofluorescence and western blotting, and the supernatant in culture medium was measured by ELISA analysis.

### Western blotting

Hippocampal tissues were extracted on the 7th day after anesthesia/surgery. Hippocampal tissues and BV2 cells were extracted with RIPA lysis buffer (Beijing Pulilai Gene Technology Co., Ltd, China), and then centrifuged at 4 °C, 12,000 g for 10 min. Protein concentration was quantified by BCA assay. The protein samples were separated by 10% sodium dodecyl sulfate–polyacrylamide gel electrophoresis and transferred electrophoretically onto a polyvinylidene fluoride membrane (Millipore). The membranes were blocked with 3%-TBST for 1 h and incubated with the following primary antibodies: NLRP3 (1:1000, df7438, Affinify, USA), ASC (1/1000, PA5-95,826, ThermoFisher, USA), IL-1β (1/1000, sc-7884, Santa Cruz, USA), Beclin-1 (1/1000, 11,306–1-ap, Proteintech, USA), and LC3-I/II (1/1000,12741T, CST, USA) at 4 °C overnight. The membrane was then washed in TBST three times (10 min each) and incubated with secondary antibody at room temperature for 2 h. An enhanced chemiluminescence system was used to detect the membrane (Chemi DocTM XRS + , China) and the results were analyzed using an imaging system (Tanon-5200, China).

### Immunofluorescence

The brain tissue slices were washed with PBS and 0.4% Triton X-100, and then, sections of brain tissue were blocked with 10% normal donkey serum for 1 h at room temperature. The brain slices were incubated with primary antibody NLRP3 (1/100, df7438, Affinify, USA). Following the treatments, cells were stained with Mito-Tracker Red CMXRos fluorescent probe for 30 min at 37 °C. BV2 cells were fixed with 4% paraformaldehyde at 37 °C for 15 min, and washed with PBS twice. Then, permeabilization with 0.5% Triton-X-100 at 37 °C for 30 min and blocking with goat serum for 1 h. Cells were incubated with antibody LC3I/II (1:250, af540, Affinity, USA). Nuclear DNA was labeled with DAPI. Fluorescent images were observed under laser confocal microscope (FV1000, Olympus, JAPAN).

### Mitochondrial membrane potential

After culturing, BV2 cells were harvested by centrifugation (3 min at 1500 × g) and then resuspended in 500 µL of incubation buffer with JC-1 (10 ug/mL) for 20 min at 37 °C and 5% CO_2_ in the dark. JC-1 is a cationic dye that reflects mitochondrial polarization by transferring fluorescence emission from green (530 nm) to red (590 nm). In flow cytometry, the green and red fluorescence signals were detected, respectively, in the conventional FL-1 and FL-2 channels. Samples were analyzed using novoexpress software. The ratio of red-to-green fluorescence measures changes in mitochondrial membrane potential (MMP).

### Enzyme-linked immunosorbent assay

IL-1β was measured in BV2 cells supernatant using an enzyme-linked immunosorbent assay (ELISA) kit following the manufacturer’s instructions (Meimian Industrial Co., Ltd, Jiangsu, China).

### Statistical analyses

Data were analyzed with GraphPad Prism 6.0 (GraphPad Software Inc., USA). Data are presented as mean ± SEM. The escape latency and average speed were analyzed by two-way analysis of variance (ANOVA); moreover, the time spent in the target quadrant by one-way ANOVA. A one-way ANOVA was used to perform the mitochondrial membrane potential, relative protein levels of Beclin-1, LC3I and LC3-II, and relative protein levels of NLRP3, caspase-1, ASC, and IL-1 β.* P* < 0.05 was considered statistically significant.

## Results

### MCC950 attenuated the negative effects of anesthesia/surgery on learning and memory

**Fig. 1:**
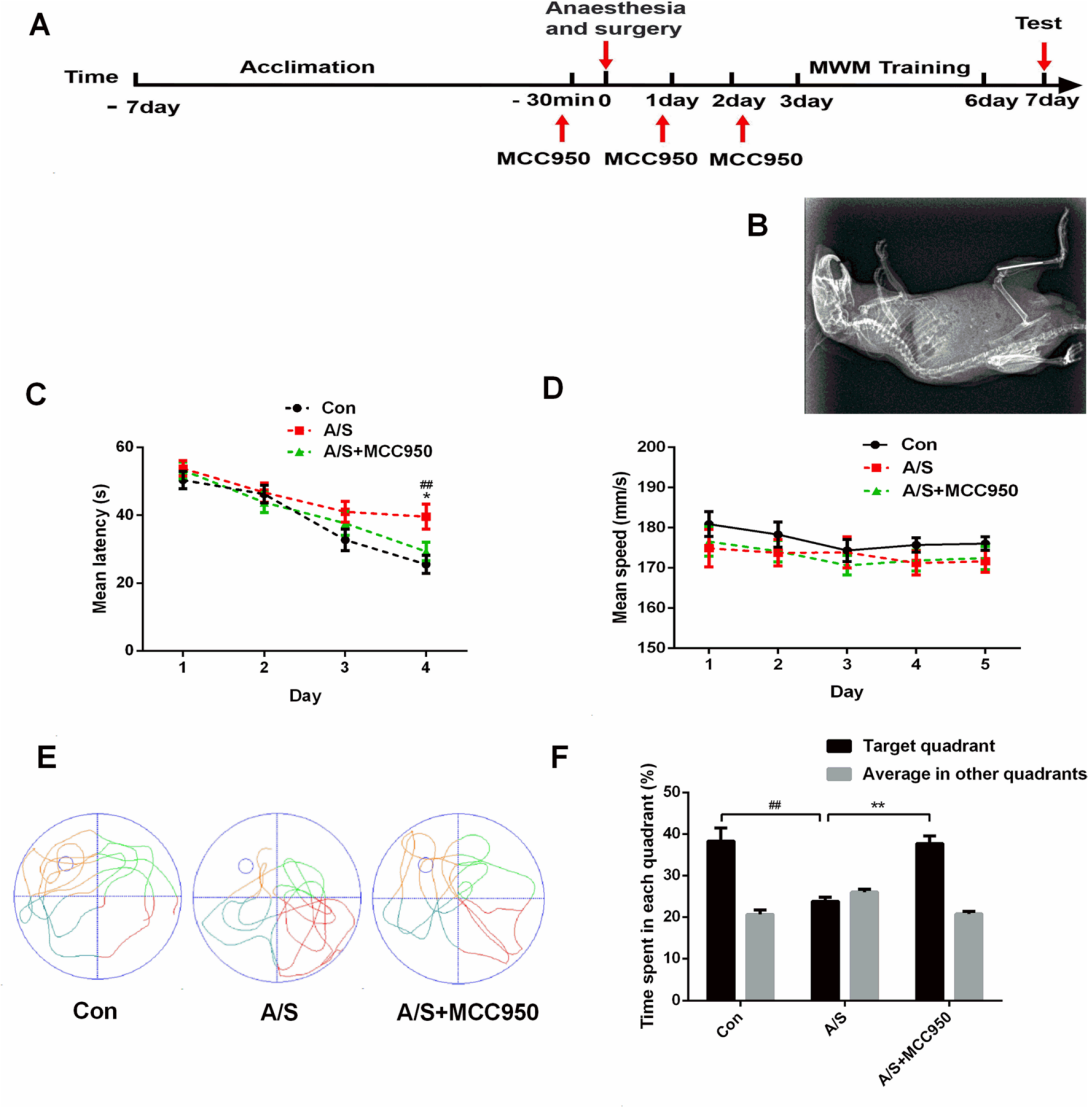
MCC950 treatment alleviated the effects of anesthesia/surgery on learning and memory in aged mice. **A** Schematic timeline of the experimental paradigm. Treatment with MCC950 before anesthesia and surgery and on days 1 and 2 after surgery. The mice rested for 2 days after anesthesia and surgery, and the Morris Water Maze was used to assess learning and memory on the third day after anesthesia and surgery. After 4 days of training, the probe test was conducted on the 7th day. **B** Tibial fracture model in elderly mice. X-ray was used to determine the intramedullary fixation of the left tibial fracture in mice. **C** Escape latency to reach the hidden platform during the 4-day training; MCC950 reversed the increased escape latency caused by anesthesia/surgery. ^##^*P* < 0.01 versus Con group; *P < 0.05 versus A/S group. **D** Average swimming speed; there was no significant difference among the three groups. **E** Representative exploratory path of mice in the probe test, each quadrant is represented in a different color, and the quadrant with the small circle is the target quadrant. **F** The percentage of time spent in the target quadrant during the probe test; MCC950 reversed anesthesia/surgery-induced reduction the time spent in the target quadrant. All data are presented as mean ± SEM (*n* = 12 per group). ^##^*P* < 0.01 versus Con group; **P < 0.01 versus A/S group. *Con* control group, *A/S* anesthesia/surgery group, *A/S + MCC950* anesthesia/surgery group combined with MCC950 treatment group

To identify the role of NLRP3 inflammasome in learning and memory dysfunction induced anesthesia/surgery, we used MWM to test the effects of the NLRP3 inhibitor MCC950 on learning and memory in elderly mice. Mice were intraperitoneally injected with MCC950 (10 mg/kg) 30 min before surgery and on days 1 and 2 after surgery. The mice acclimated to the environment for 7 days before the experiment began, as shown in Fig. 1A. Tibial fracture with intramedullary fixation was performed under isoflurane anesthesia. The mice rested for 2 days after anesthesia and surgery, and the MWM was used to assess learning and memory on the third day after anesthesia and surgery (Fig. 1A); tibial fracture model in elderly mice (Fig. 1B). Compared with the control group, the escape latency was significantly prolonged on the fourth day of training test in the anesthesia/surgery group (F (2, 132) = 5.275, *P* < 0.01, Fig. 1C). There was no significant difference in swimming speed (F (2, 165) = 1.600, *P* > 0.05; Fig. 1D) among the three groups. Exploratory path of three groups of mice in the probe test (Fig. 1E). The percentage of time spent in the target quadrant was more in the control group than the anesthesia/surgery group (F (2, 66) = 4.809, *P* < 0.01, Fig. 1F). Interestingly, all of the changes in the behavioral tests were reversed by administration of MCC950 (F (2, 132) = 5.275, *P* < 0.01; F (2, 66) = 5.616, *P* < 0.01, Fig. 1C, F). These data indicated that MCC950 treatment had a therapeutic effect on anesthesia/surgery-induced cognitive decline.

### Administration of MCC950 inhibited NLRP3 inflammasome activation induced by anesthesia and surgery in the hippocampus of aged mice

**Fig. 2:**
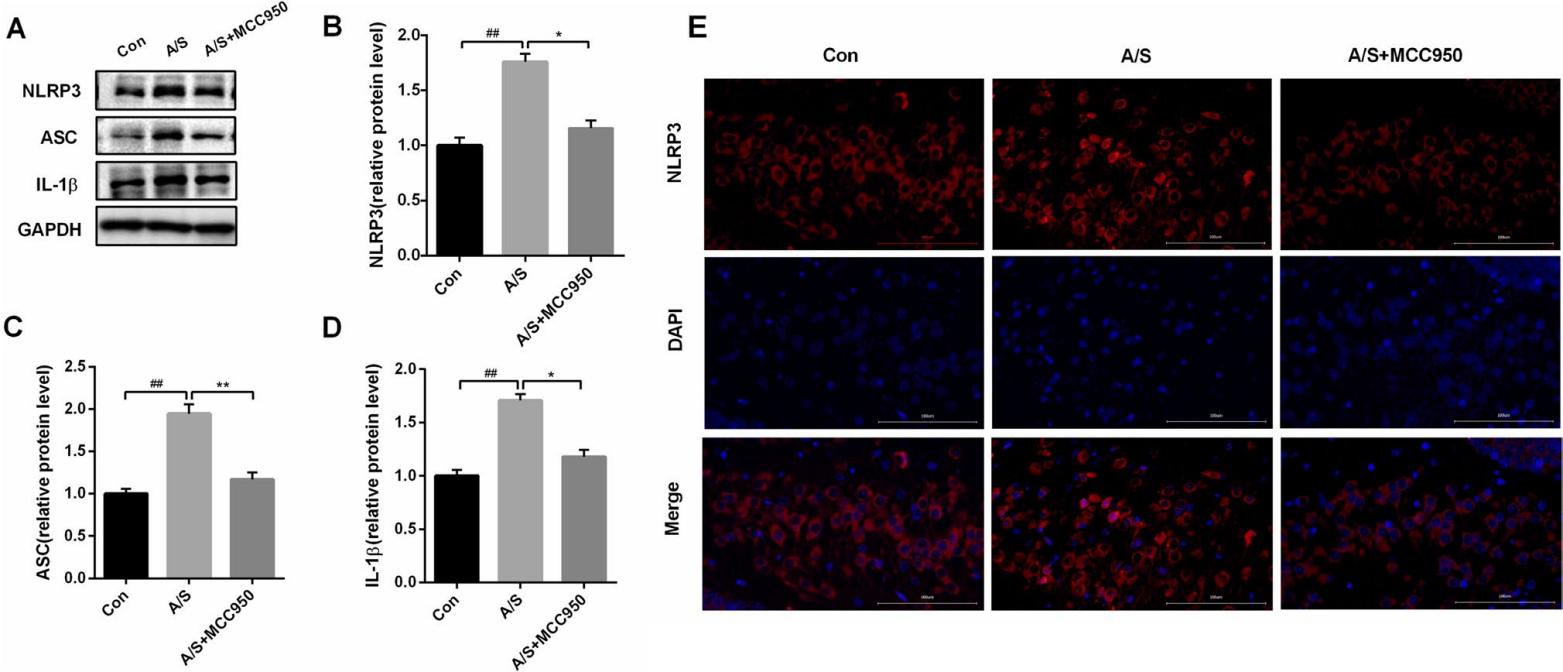
Effect of MCC950 treatment on NLRP3 inflammasome in the hippocampus of aged mice. **A** Representative western blot illustrating NLRP3, ASC, and IL-1β levels in the hippocampus on the 7th day after anesthesia/surgery. **B**–**D** MCC950 reversed anesthesia/surgery-induced increase in NLRP3, ASC, and IL-1β levels in the hippocampus on the 7th day after anesthesia/surgery. The data are presented as means ± SEM (*n* = 6 per group). ^##^*P* < 0.01 versus Con group; **P < 0.01, *P < 0.05 versus A/S group. **E** Representative images of immunofluorescence staining of NLRP3 (red) in the hippocampus. Scale bars = 100 μm. *Con* control group, *A/S* anesthesia/surgery group, *A/S + MCC950* anesthesia/surgery group combined with MCC950 treatment group

To determine the role of NLRP3 inflammasome in learning and memory impairment induced by anesthesia and surgery, the present study examined the effect of MCC950 on the levels of NLRP3 inflammasome components in the hippocampus. Compared with the control group, the expression of NLRP3 (F = 33.06, *P* < 0.01), ASC (F = 34.56, *P* < 0.01), and IL-1β (F = 36.09, *P* < 0.01) was significantly increased in the hippocampus at day 7 post-surgery in the anesthesia/surgery group by western blotting (Fig. 2A–D). However, administration of MCC950 effectively reduced the anesthesia/surgery-induced over-expression of NLRP3, ASC, and IL-1β in the hippocampus (Fig. 2A–D). Meanwhile, immunofluorescence staining of NLRP3 in the hippocampus was confirmed (Fig. 2E). Overall, these results indicated that anesthesia/surgery-induced NLRP3 inflammasome activation was remarkably relieved by MCC950 administration.

### Mitophagy inhibited LPS-induced activation of NLRP3 inflammasome in BV2 cells

**Fig. 3:**
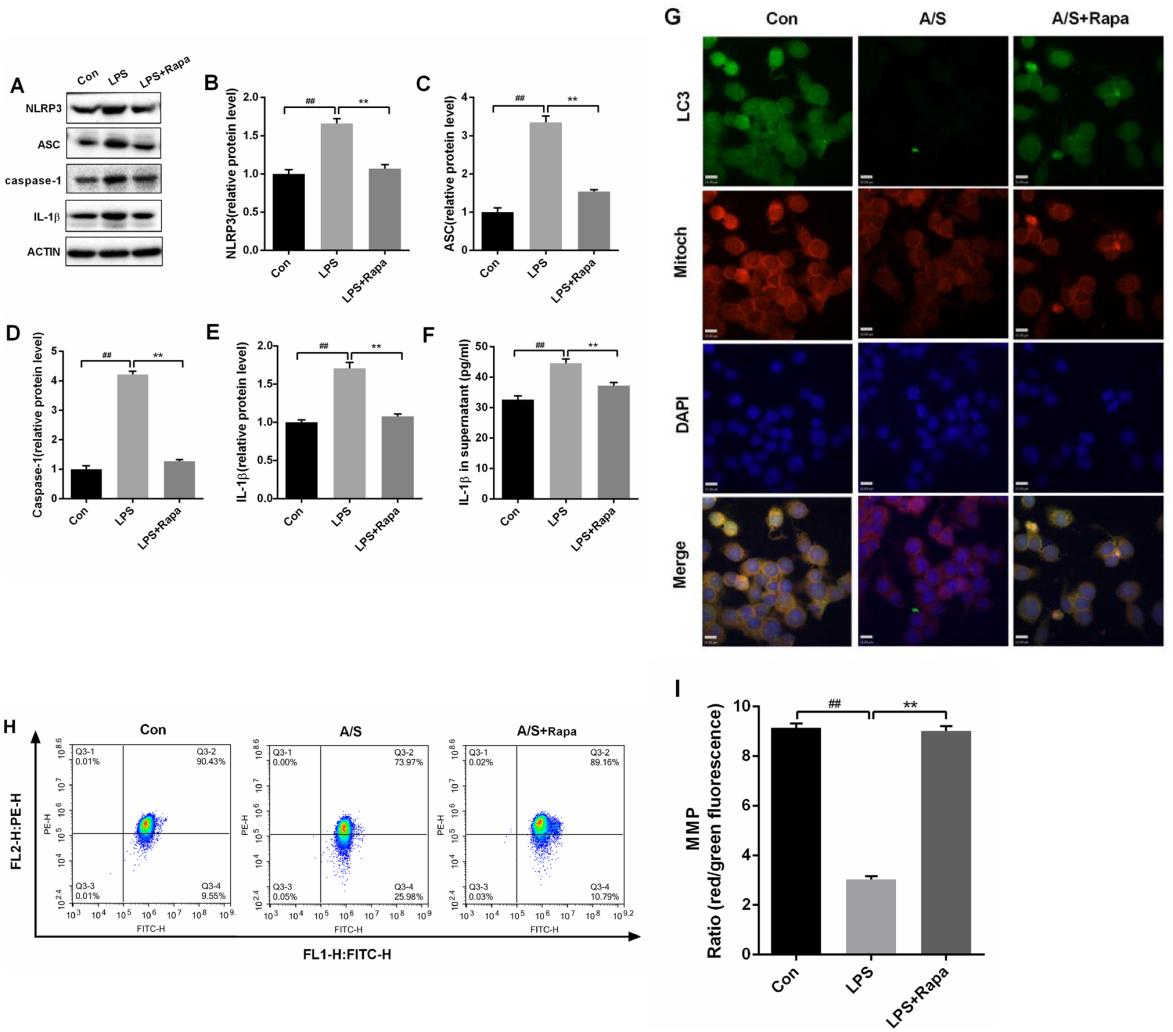
Mitophagy inhibited the activation of NLRP3 inflammasome in activated BV-2 microglial cells. **A** Representative western blot illustrating NLRP3, ASC, caspase-1, and IL-1β levels in BV2 cells after LPS stimulation (1 μmol/L), Rapamycin (Rapa) treatment (0.1 μmol/L) for 24 h. **B**–**E** Rapamycin reversed LPS-induced increase in NLRP3, ASC and IL-1β levels in BV2 cells. **F** ELISA assays of IL-1β levels in the supernatant of BV2 cells. The data are presented as means ± SEM (*n* = 6 per group). ^##^*P* < 0.01 versus Con group; **P < 0.01 versus A/S group. **G** LC3 levels in the mitochondria were determined by immunofluorescence. Mitochondria were stained with Mito-Tracker Red, and Nuclei are stained with DAPI. The merged images show the Nuclei (blue), LC3 (green), and Mito-Tracker (red) signals. Scale bars = 15 μm. **H** The mitochondrial membrane potential (MMP) was measured using a JC-1 probe. When the membrane potential was normal, it was red fluorescence by flow detection. While decreased, it was green fluorescence. **I** The ratio of red fluorescence to green fluorescence reflects the mitochondrial membrane potential (MMP). Data are mean ± SEM (*n* = 6 per group). ^##^*P* < 0.01 versus Con group; **P < 0.01 versus A/S group. *Con* control group, *LPS* LPS group, *LPS + Rapa* LPS group combined with Rapamycin treatment

To further study the role of mitophagy in regulating NLRP3 inflammasome, we established LPS-stimulated BV-2 cell model. Mitophagy inducer with Rapamycin was used to investigate the expression of mitophagy markers and NLRP3 inflammasome. Our data showed that the expression of NLRP3 (F = 39.69, *P* < 0.01), ASC (F= 106.7, *P* < 0.01), caspase-1 (F=324.4, *P* < 0.01), and IL-1 β (F =55.77, *P* < 0.01) were significantly higher in LPS-stimulated BV-2 microglial cells than normal BV-2 microglial cells by western blotting (Fig. 3A–E). Meanwhile, immunofluorescence staining of NLRP3 in BV2 cells was confirmed (Fig. 3G). The levels of IL-1β in the supernatant of cells were significantly higher in LPS-stimulated BV-2 microglial cells than normal BV-2 microglial cells (F = 24.91, *P* < 0.01, Fig. 3F). Mitochondrial membrane potential (MMP) was lower in LPS-stimulated BV-2 microglial cells than normal BV-2 microglial cells (F = 405.1, *P* < 0.01, Fig. 3H,I). Administration of Rapamycin reduced the over-expression of NLRP3 (F = 39.69, *P* < 0.01), ASC (F = 106.7, *P* < 0.01), caspase-1 (F = 324.4, *P* < 0.01), and IL-1 β (F = 55.77*,* *P* < 0.01) in LPS-stimulated BV-2 microglial cells, while increased MMP (F = 405.1, *P* < 0.01, Fig. 3A–F, H, I). In summary, the results showed that NLRP3 inflammasome activation was dramatically relieved by Rapamycin administration in LPS-stimulated BV-2 microglial cells. Nevertheless, whether mitophagy induction can effectively inhibit the activation of NLRP3 inflammasome needs to be further explored in vivo.

### Anesthesia and surgery-induced NLRP3 inflammasome activation in aged brain by inhibiting mitophagy

**Fig. 4:**
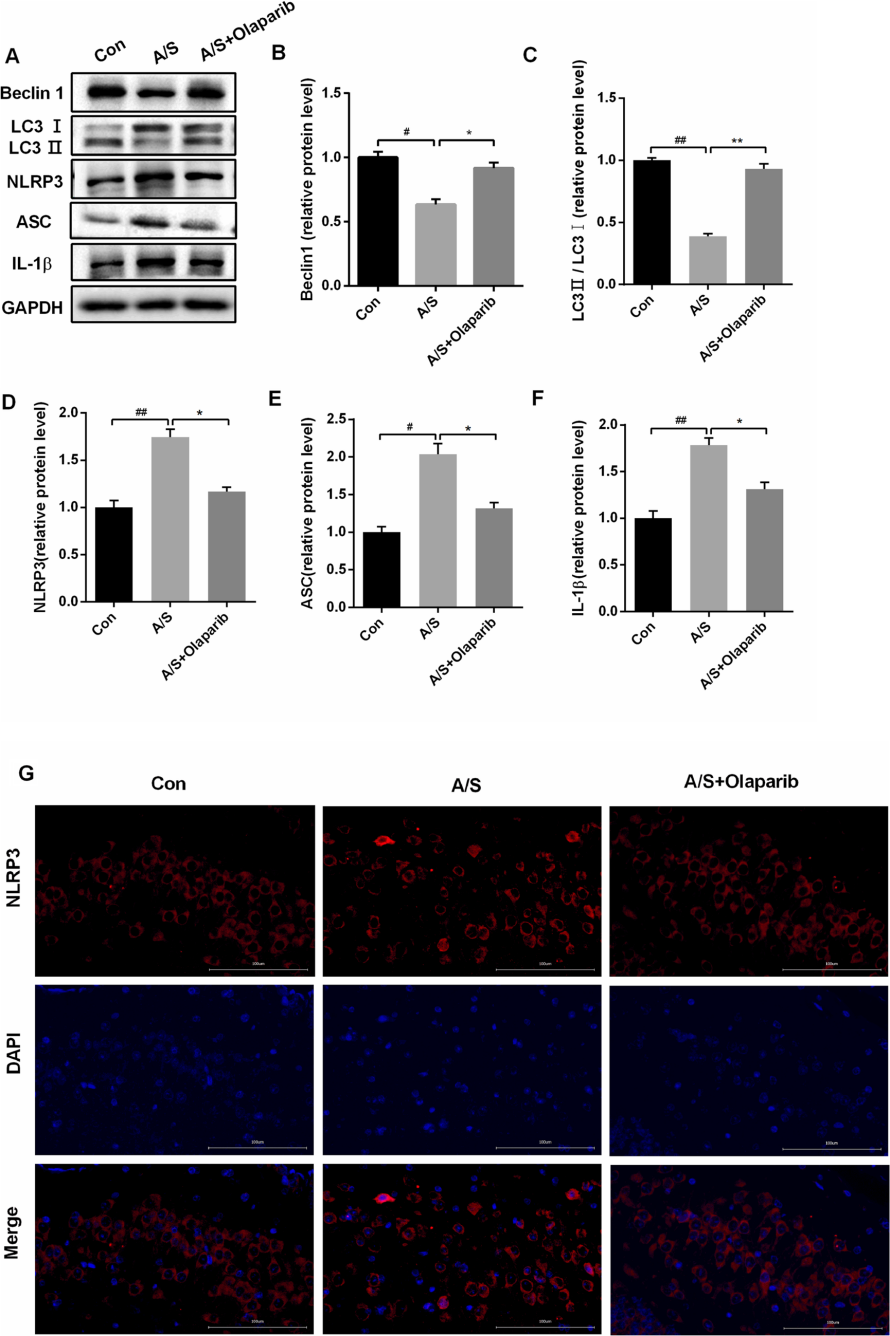
Olaparib treatment inhibited the activation of NLRP3 inflammasome and increased LC3-II and Beclin1 levels in the hippocampus of aged mice. **A** Representative western blot illustrating the effects of Olaparib on the anesthesia/surgery-induced changes in hippocampal NLRP3, ASC, IL-1β, and LC3-II, Beclin1 levels on the 7th day after anesthesia/surgery. **B**–**F** Olaparib reversed anesthesia/surgery-induced decrease in Beclin1, LC3-II, and increase in NLRP3, ASC, and IL-1β levels in the hippocampus on the 7th day after anesthesia/surgery. The data are presented as means ± SEM (*n* = 6 per group). ^##^*P* < 0.01, ^#^*P* < 0.05 versus Con group; **P< 0.01, *P < 0.05 versus A/S group. **G** Representative images of immunofluorescence staining of NLRP3 (red) in the hippocampus. Scale bars = 100 μm *Con* control group; *A/S* anesthesia/surgery group; *A/S + Olaparib* anesthesia/surgery combined with Olaparib treatment group

To further clarify whether the inhibition of mitophagy is involved in the anesthesia/surgery-induced NLRP3 inflammasome activation, mitophagy activation with Olaparib was used to investigate the expression of mitophagy markers and NLRP3 inflammasome. The results showed that compared with the control group, the expression of NLRP3 (F = 32.17, *P* < 0.01), ASC (F = 26.01, *P* < 0.01), and IL-1β (F = 28.48, *P* < 0.01) was significantly increased, while the expression of LC3II/I (F = 129.5, *P* < 0.01) and Beclin1 (F = 19.26, *P* < 0.01) decreased in the hippocampus at day 7 post-surgery in the anesthesia/surgery group (Fig. 4A–F). Meanwhile, immunofluorescence staining of NLRP3 was confirmed in the hippocampus (Fig. 4G). Administration of Olaparib reduced the anesthesia/surgery-induced over-expression of NLRP3 (F = 32.17, *P* < 0.01), ASC F = 26.01, *P* < 0.01) and IL-1β (F = 28.48, *P* < 0.01) in the hippocampus; however, the expression of LC3II/I (F = 129.5, *P* < 0.01) and Beclin1 (F = 19.26, *P* < 0.01) was upregulated (Fig. 4A–F). Taken together, the results showed that mitophagy played an important role in inhibition of NLRP3 inflammasome activation induced by anesthesia/surgery.

### Mitophagy activation with Olaparib reversed anesthesia/surgery-induced learning and memory dysfunction in aged mice

**Fig. 5:**
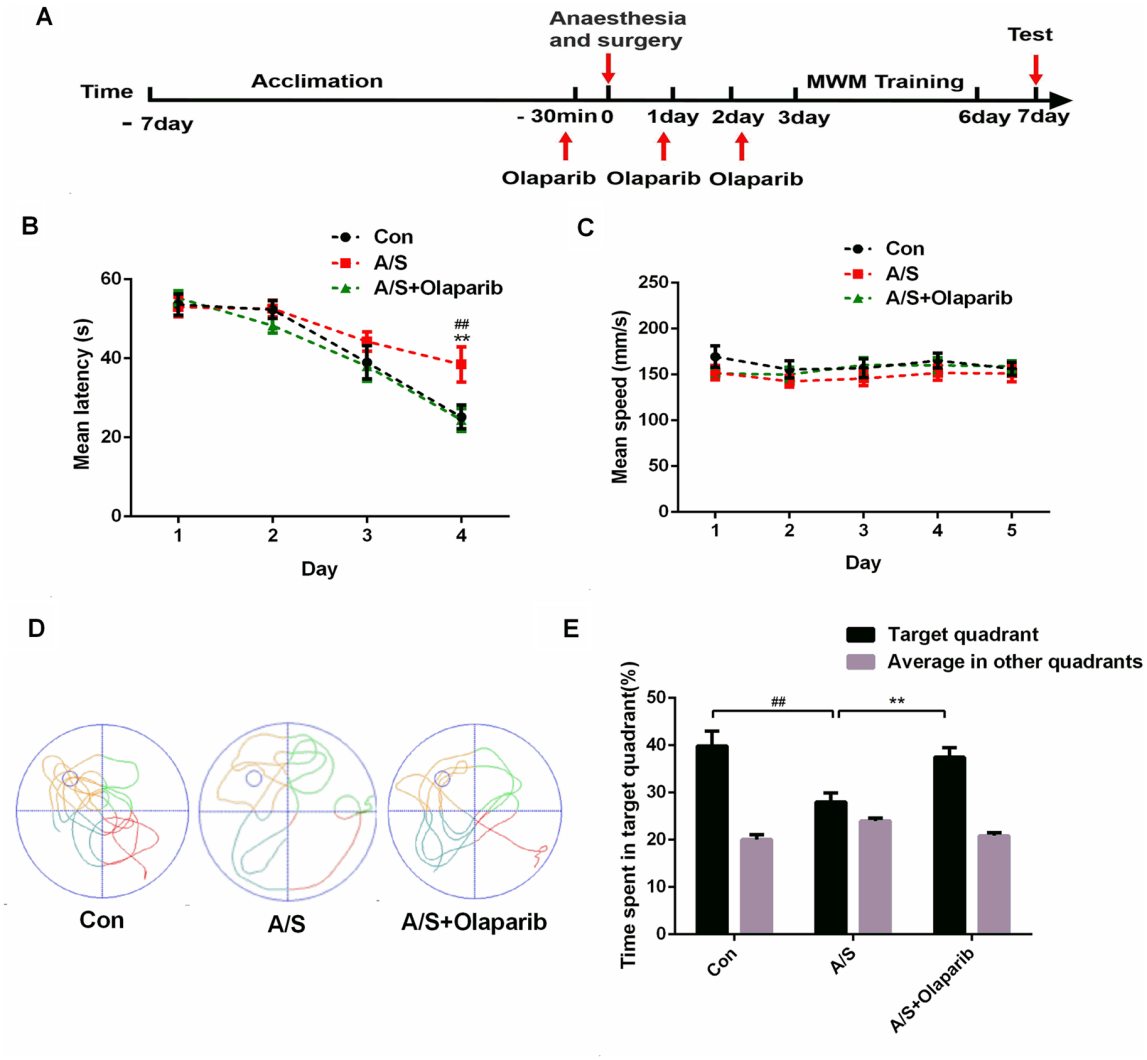
Olaparib treatment alleviated the effects of anesthesia/surgery on learning and memory in aged mice. **A** Schematic timeline of the experimental paradigm. Treatment with Olaparib before anesthesia and surgery and on days 1 and 2 after surgery, after 4 days of training, the probe test was conducted on the 7th day. **B** Escape latency to reach the hidden platform during the 4-day training; Olaparib reversed the increased escape latency caused by anesthesia/surgery. ^##^*P* < 0.01 versus Con group; **P < 0.01 versus A/S group. **C** Average swimming speed; there was no significant difference among the three groups. **D** Representative exploratory path of mice in the probe test. **E** The percentage of time spent in the target quadrant during the probe test; Olaparib reversed anesthesia/surgery-induced reduction the time spent in the target quadrant. All data are presented as mean ± SEM (*n* = 12 per group). ^##^*P* < 0.01 versus Con group; **P < 0.01 versus A/S group. *Con* control group; *A/S* anesthesia/surgery group; *A/S + Olaparib* anesthesia/surgery combined with Olaparib treatment group

To identify the role of mitophagy in learning and memory dysfunction induced anesthesia/surgery, we used the MWM test to explore the effects of Olaparib, a mitochondrial autophagy activator, on learning and memory function. Mice were intraperitoneally injected with Olaparib (10 mg/kg) 30 min before surgery and on days 1 and 2 after surgery. Mice acclimated to the environment for 7 days before the experiment began, as shown in Fig. 5A. Tibial fracture with intramedullary fixation was performed under isoflurane anesthesia. The mice rested for 2 days after anesthesia and surgery, and the MWM was used to assess learning and memory on the third day after anesthesia and surgery (Fig. 5A). There was no significant difference in swimming speed (F (2, 165) = 2.716, *P* > 0.05, Fig. 5C) among the three groups. Compared with the control group, the escape latency was significantly prolonged on the fourth day of training test (F (2, 132) = 4.942, *P* < 0.01, Fig. 5B) as well as target quadrant time (F = 6.831, *P* < 0.01, Fig. 5E) in the probe trial was significantly decreased in the anesthesia/surgery group. Of note, all of the changes in the behavioral tests (F (2, 132) = 4.942, *P* < 0.01; F = 6.831, *P* < 0.01, Fig. 5B, E) were reversed by administration of Olaparib. Exploratory path of three groups of mice in the probe trial (Fig. 5D). Taken together, these data indicated that Olaparib treatment had a therapeutic effect on the cognitive impairment induced by anesthesia/surgery.

## Discussion

In our study, we found that older mice had poorer learning and memory function after anesthesia and surgery, and anesthesia and surgery did not impair learning and memory abilities in young mice (Zhao et al. 2016). In addition, we found that anesthesia and surgery led to overactivation of NLRP3 inflammasome, decreased the levels of mitophagy-related proteins, including Beclin1, LC3II, and induced mitochondria dysfunction in the hippocampus of aged mice. Conversely, Olaparib, a mitophagy inducer, enhanced mitophagy, improved mitochondrial health, inhibited NLRP3 inflammasome activation, and ameliorated the learning and memory deficits in aged mice. Overall, our results suggest that mitophagy may play a vital role in NLRP3 inflammasome activation-mediated cognitive impairment after anesthesia and surgery in aged mice. Thus, this present study suggests that mitophagy inducer can restore cognitive decline caused by anesthesia/surgery.

Morris Water Maze is a common cognitive function test method, which can objectively reflect learning and memory abilities (Morris et al. 1982). The average swimming speed during the training and probe trials was comparable in all the mice; this reduced interference to the test results. In this study, the learning ability of mice was determined by escape latency, and the memory ability of mice was determined by time spent in the target quadrant. Our results showed that during the training trials, mice in the anesthesia/surgery group showed a longer escape latency than mice in the control group. During the probe trials, mice in the anesthesia/surgery group showed significantly less preference for the target quadrant than control mice. The results showed that anesthesia/surgery-induced learning and memory impairment in older mice. Olaparib, a mitophagy inducer significantly compromised the decreased preference for target quadrant and the increase of the escape latency caused by anesthesia/surgery. Together, the results implied that anesthesia and surgery induced mitophagy dysfunction, which was associated with postoperative cognitive impairment.

The role of the NLRP3 inflammasome in POCD has recently been investigated (Wei et al. 2019; Sun et al. 2022). The NLRP3 inflammasome activation leads to secreting inflammatory factors, like IL-1β and IL-18. Overproduction of IL-1β and IL-18 has been reported to be involved in systemic inflammation (Sendler et al. 2020). In the present study, enhanced NLRP3 inflammasome activation were detected after anesthesia and surgery, and NLRP3 inhibition with MCC950 significantly inhibited NLRP3 inflammasome-mediated caspase-1 and IL-1β maturation.

Several theories have been proposed to explain activation of the NLRP3 inflammasome, including reactive oxygen species (ROS) production and mitochondrial DNA (mtDNA) release (Lamkanfi and Dixit 2014). Qiu et al. showed that ROS are positively related to postoperative cognitive deficit and mitochondria is thought to be the main source of intracellular ROS (Qiu et al. 2016a, b). ROS overproduction can activate NLRP3 inflammation (Qiu et al. 2016a, b). Oxidative stress caused by surgical trauma leads to increased mitochondrial permeability, and mtDNA is released into the cytoplasm, causing activation of NLRP3 inflammasome (Zhao et al. 2021). These existing findings suggest that NLRP3 inflammasome activation is enhanced due to mitochondrial dysfunction, such as excessive mitochondrial ROS and change in mitochondrial membrane permeability. However, mitophagy is the main mechanism of dysfunctional mitochondrial clearance and controls mitochondrial quality (Harris et al. 2018; Zhong et al. 2016; Yamano and Tanaka 2016), thereby preventing excessive inflammation activation. In this study, we showed that anesthesia and surgery induced activation of NLRP3 inflammasome, mitochondria impairment, and mitophagy dysfunction in the hippocampus. Furthermore, we showed that Olaparib relieved the anesthesia/surgery-induced mitochondria impairment. Therefore, Olaparib increased activating mitophagy in parallel with inactivation of the NLRP3 inflammasome. Therefore, our findings indicated that mitophagy-mediated inhibition of the NLRP3 inflammasome was associated with improvement in anesthesia/surgery-induced cognitive impairment. Chang et al. showed that resveratrol inhibited NLRP3 inflammasome activation in macrophages by preserving mitochondrial integrity and enhancing autophagy (Chang et al. 2015). Qiu et al. reported that Urolixin A inhibited NLRP3 inflammasome activation via promoting mitophagy in microglia, and improved dopaminergic neurodegeneration and neuroinflammation (Qiu et al. 2022). Shao et al. showed that Divanillyl sulfone suppressed NLRP3 inflammasome activation by inducing mitophagy in microglia and ameliorates chronic neuropathic pain in mice (Shao et al. 2021). Zheng et al. showed that FUN14 domain containing 1(FUNDC1) inhibited NLRP3 inflammasome activation by promoting mitophagy, thereby alleviated intracerebral hemorrhage-induced brain injury (Zheng et al. 2021). These studies also suggested that alleviating NLRP3-mediated neuroinflammation by promoting mitophagy plays an important role in the diseases of central nervous system.

The current study does have limitations. First, we did not identify specific mechanism by which mitophagy inhibited NLRP3 inflammasome activation in anesthesia/surgery-induced cognitive impairment. Future research is needed to determine the specific molecular mechanisms. Second, Olaparib, a mitophagy inducer, was observed and tested only for 7 days after anesthesia/surgery. The long-term effects of enhancing mitophagy on anesthesia/surgery-induced learning and memory decline need to be further investigated.

In conclusion, the data presented here revealed that activating mitophagy inhibited anesthesia/surgery-induced learning and memory decline in the older mice by promoting NLRP3 inflammasome inactivation, which reduced IL-1β secretion. These results show that enhancing mitophagy may be further developed as a potential anti-inflammatory agent for the treatment of POCD.

## Data Availability

The original contributions presented in the study are included in the article, and further inquiries can be directed to the corresponding author.
